# Parkinson’s Disease-Specific Autoantibodies against the Neuroprotective Co-Chaperone STIP1

**DOI:** 10.3390/cells11101649

**Published:** 2022-05-16

**Authors:** Jolene Su Yi Tan, Bernett Lee, Jackwee Lim, Dong Rui Ma, Jia Xin Goh, Suh Yee Goh, Muhammad Yaaseen Gulam, Ser Mei Koh, Weiling Wendy Lee, Lei Feng, Qing Wang, Yinxia Chao, Olaf Rötzschke, Eng King Tan

**Affiliations:** 1 Neuroscience and Behavioural Disorders Department, Duke-NUS Medical School, Singapore 169857, Singapore; e0011424@u.duke.nus.edu (J.S.Y.T.); chao.yinxia@singhealth.com.sg (Y.C.); 2 Department of Neurology, Singapore General Hospital, Singapore 169608, Singapore; ma.dong.rui@sgh.com.sg (D.R.M.); goh.jia.xin@sgh.com.sg (J.X.G.); goh.suh.yee@sgh.com.sg (S.Y.G.); muhd.yaaseen.gulam.mohd@sgh.com.sg (M.Y.G.); 3 National Neuroscience Institute, Tan Tock Seng Hospital, Singapore 308433, Singapore; 4 Singapore Immunology Network (SIgN), A*STAR, Singapore 138648, Singapore; bernett_lee@immunol.a-star.edu.sg (B.L.); lim_jack_wee@immunol.a-star.edu.sg (J.L.); koh_ser_mei@immunol.a-star.edu.sg (S.M.K.); wendy_lee@immunol.a-star.edu.sg (W.W.L.); olaf_rotzschke@immunol.a-star.edu.sg (O.R.); 5 Centre for Health Longevity, National University Hospital, Singapore 119074, Singapore; fenglei2020sg@nus.edu.sg; 6 Department of Psychological Medicine, Yong Loo Lin School of Medicine, National University of Singapore, Singapore 119077, Singapore; 7 Healthy Longevity Translational Research Programme, Yong Loo Lin School of Medicine, National University of Singapore, Singapore 119077, Singapore; 8 Department of Neurology, Zhujiang Hospital, Southern Medical University, Guangdong 510282, China; wqdennis@hotmail.com

**Keywords:** Parkinson’s disease, autoantibodies, STIP1, autoimmunity, neurodegeneration

## Abstract

Parkinson’s disease (PD) is a debilitating movement disorder characterised by the loss of dopaminergic neurons in the substantia nigra. As neuroprotective agents mitigating the rate of neurodegeneration are unavailable, the current therapies largely focus only on symptomatic relief. Here, we identified stress-inducible phosphoprotein 1 (STIP1) as a putative neuroprotective factor targeted by PD-specific autoantibodies. STIP1 is a co-chaperone with reported neuroprotective capacities in mouse Alzheimer’s disease and stroke models. With human dopaminergic neurons derived from induced pluripotent stem cells, STIP1 was found to alleviate staurosporine-induced neurotoxicity. A case-control study involving 50 PD patients (average age = 62.94 ± 8.48, Hoehn and Yahr >2 = 55%) and 50 age-matched healthy controls (HCs) (average age = 63.1 ± 8) further revealed high levels of STIP1 autoantibodies in 20% of PD patients compared to 10% of HCs. Using an overlapping peptide library covering the STIP1 protein, we identified four PD-specific B cell epitopes that were not recognised in HCs. All of these epitopes were located within regions crucial for STIP1’s chaperone function or prion protein association. Our clinical and neuro-immunological studies highlight the potential of the STIP1 co-chaperone as an endogenous neuroprotective agent in PD and suggest the possible involvement of autoimmune mechanisms via the production of autoantibodies in a subset of individuals.

## 1. Introduction

Parkinson’s disease is one of the most common neurodegenerative movement disorders. By 2050, Parkinson’s disease is estimated to affect more than 12 million individuals worldwide [1]. It is neuropathologically characterised by the loss of dopaminergic neurons in the substantia nigra pars compacta (SNc) and the formation of Lewy Body inclusions [2]. Clinical diagnosis is based on the manifestation of motor deficits consisting of resting tremor, rigidity, and bradykinesia. On top of these motor deficits, patients experience a range of prodromal non-motor symptoms such as autonomic dysfunctions, cognitive impairment, sleep disturbances, and psychiatric disorders [3]. At the time of motor symptom onset, approximately 30% to 68% of dopaminergic neurons in the SNc are lost [2,4]. Despite decades of research on disease modifying therapies in Parkinson’s disease, the current treatment of Parkinson’s disease only provides symptomatic relief. The search for agents capable of neuronal protection, rescue, and restoration remains elusive.

Here, we identified stress-inducible phosphoprotein 1 (STIP1) as a potential endogenous neuroprotective factor in Parkinson’s disease. STIP1, also known as the heat shock cognate 70/heat shock protein (HSP) 90-organising protein (Hop) or Sti1, is a well-studied co-chaperone that facilitates client protein transfer from HSP70 to HSP90. It is ubiquitously expressed in most tissues where it typically localises in the cytoplasm [5]. However, STIP1 can also be secreted into the extracellular space. Astrocytes and microglia release STIP1 via microvesicles [6,7]. Ovarian cancer tissues have also been reported to secrete STIP1, enabling the use of STIP1 levels as a prognostic disease biomarker [8].

Elevated secretion of STIP1 by astrocytes was observed under ischemic insult as well as in the brains of Alzheimer’s disease patients [9]. Extracellular STIP1 interacts with various receptors to enhance neuronal resilience, induce differentiation, cellular proliferation, and protein synthesis. Binding of STIP1 to the prion protein (PrP^C^) was discovered to promote neuritogenesis through the activation of the extracellular signal-regulated kinase 1 and 2 (ERK1/2). This binding also enabled neuroprotection through the cyclic adenosine monophosphate (cAMP)-dependent protein kinase A (PKA) pathway activation [10]. In addition, engagement of PrP^C^-STIP1 ameliorated staurosporine-induced neurotoxicity in primary hippocampal neurons and anisomycin-induced cell death in retinal neurons [11,12]. In Alzheimer’s disease models, STIP1 inhibited β-amyloid binding to PrP^C^, attenuating β-amyloid-induced neurotoxicity in mouse primary hippocampal neurons [13,14].

As seen from the presence of embryonic lethality by E10.5 in STIP1 knockout mouse models, STIP1 is pivotal in development [9]. In mice, reduced STIP1 levels led to hyperactivity and attention deficits. This further supports STIP1’s role in the development of brain circuitry, preventing the autism spectrum disorder (ASD)-like phenotype [15]. Hypomorphic expression of the STIP1 allele in mice resulted in age-dependent hippocampal neurodegeneration and a reduction in the hippocampal volume, causing deficits in spatial memory [16]. The presence of STIP1 autoantibodies in the mothers of children with ASD and patients with neuro-Bechet’s disease presents the possibility of autoimmune mechanisms against the STIP1 protein [17,18].

Although STIP1 has been thoroughly investigated to be neuroprotective in several neurodegenerative conditions, its role in Parkinson’s disease remains unknown. Hence, we sought to characterise the role of STIP1 on dopaminergic neurons and determine whether immune dysregulation in the presence of autoantibodies may predispose individuals to Parkinson’s development. Here, we provide the first evidence that STIP1’s neuroprotective effect also extends to dopaminergic neurons. Moreover, the analysis of the plasma samples revealed STIP1-specific autoantibodies that appeared to be associated with the manifestation of Parkinson’s disease. These B cell epitopes overlapped with the sites vital for its co-chaperone function and PrP^C^ engagement, with the latter also containing epitopes for autoreactive T cells. Therefore, our paper suggests the involvement of an autoimmune component in a subset of Parkinson’s disease patients.

## 2. Materials and Methods

### 2.1. Study Design

This study comprises two main sections. First, the neuroprotective effect of STIP1 was examined through the in vitro characterisation of the STIP1 protein on dopaminergic neurons. Second, we evaluated the prevalence of STIP1 autoantibodies in the Parkinson’s disease patients and healthy controls (HCs) to determine whether it has an impact on disease manifestation. Following the identification of high STIP1 autoantibodies in a subset of patients and HCs, we performed a detailed characterisation of the B and T cell epitopes using the STIP1 peptide-based ELISA and T cell elispot, respectively. This is an exploratory study with no prior work conducted. The outcomes include the recruitment of 50 Parkinson’s disease patients and 50 HCs. The predictor refers to the measured STIP1 autoantibody levels. Potential confounders such as age and gender were accounted for while other neurodegenerative conditions and specific co-morbidities that included ASD, malignancy, and autoimmune conditions were excluded.

### 2.2. Recruitment of Parkinson’s Disease Patients and Healthy Controls

This study was performed between August 2019 and September 2021. Parkinson’s disease patients (*n* = 50) diagnosed and examined by movement disorder neurologists at tertiary referral centres were recruited. The diagnosis of Parkinson’s disease was based on the United Kingdom Parkinson’s Disease Society Brain Bank clinical diagnostic criteria without postmortem pathology examination [19]. Severity was assessed using the Hoehn and Yahr staging. Healthy individuals who matched the age and gender demographics of the Parkinson’s disease patients were included as controls (*n* = 50). Subjects with evidence of other neurodegenerative diseases were excluded. Individuals identified to have high STIP1 autoantibodies were recalled for further characterisation of STIP-specific T cells. Written and signed informed consent forms were obtained from all participants according to the tenets of the Declaration of Helsinki. The study was approved by the Singhealth Institutional Review Board.

### 2.3. Blood Processing and Generation of Dopaminergic Neurons Derived from Induced Pluripotent Stem Cells

Peripheral blood mononuclear cells (PBMCs) and plasma were isolated from fresh human venous blood and cryopreserved. The generation of dopaminergic neurons derived from human-induced pluripotent stem cells (hiPSCs) was performed by reprogramming PBMCs as previously described [20]. Briefly, human PBMCs lysed in RBC buffer were reprogrammed using the OCT4, SOX2, KLF4, and cMYC Sendai virus (CytoTune-iPS Reprogramming Kit, ThermoFisher Scientific, Tokyo, Japan) with a multiplicity of infection of 5 after expansion. The hiPSCs colonies with an embryonic stem cell-like appearance were manually identified and isolated D18–25 post infection. All hiPSC clones were screened for pluripotency and stable karyotypes using the G-banding chromosomal analysis. Samples used for reprogramming were approved under study number CIRB 2018/2920.

For differentiation into dopaminergic neurons, hiPSCs were dissociated with Accutase (Invitrogen, Carlsbad, CA, USA) and plated on a growth factor reduced Matrigel (BD Bioscience, Bedford, MA, USA) in the presence of 10 ng/mL fibroblast growth factor (FGF) 2 (Peprotech, Rocky Hill, NJ, USA). After 72 h, media containing 50 ng/mL Noggin (Peprotech), 10 μM SB431542 (Tocris Bioscience, Bristol, UK), and 2 μM Dorsomorphin (Tocris Bioscience) were used on the first day. Supplementation with 200 ng/mL SHH C24II (R&D Systems, Minneapolis, MN, USA) and 50 ng/mL Wnt1 (Peprotech) was performed on the second day. After 5 days, cross-tapering of the media was conducted using N2B27 media (STEMCELL Technologies, Vancouver, BC, Canada) containing the aforementioned ligands over 7 days. Cells were then maintained in N2B27 media with 200 ng/mL SHH C24II, 20 ng/mL BDNF (Peprotech), 0.2 mM ascorbic acid (Sigma, St. Louis, MO, USA), and 100 ng/mL FGF8 (Peprotech). Further supplementation with 10 ng/mL glial cell line-derived neurotrophic factor (GDNF) (Peprotech), 1 ng/mL TGFβ3 (Peprotech), and 0.5 mM dibutyryl-cAMP (Merck, Darmstadt, Germany) over 7 days was conducted for neuronal maturation.

### 2.4. Cell Culture and Neuronal Differentiation

The human neuroblastoma cell line, SH-SY5Y cells (ATCC CRL-2266, Manassa, VA, USA), were cultured in Dulbecco’s modified Eagle’s medium nutrient mixture F12 Ham media (DMEM-F12) (Lonza, Basel, Switzerland) supplemented with 10% heat inactivated fetal bovine serum (Lonza), penicillin (100 units/mL), and streptomycin (100 units/mL) (Gibco, Waltham, MA, USA) at 37 °C in a humidified incubator with 5% CO_2_. Cells were cultured in T75 tissue culture flasks (SPL Life Sciences, Gyeonggi-do, Korea) and passaged every 3 days with trypsin/ethylenediaminetetraacetic acid (Gibco).

The Lund human mesencephalic (LUHMES) cells (ATCC CR-2927) were seeded on 50 μg/mL poly-L-ornithine (Merck) and 1 μg/mL human plasma fibronectin (Merck) pre-coated plates. Cells were cultured in DMEM-F12 media supplemented with 1 × N2 supplement (Gibco), 0.5 mM sodium pyruvate (Gibco), and 40 ng/mL human recombinant basic FGF (R&D systems, Minneapolis, MN, USA). Cell differentiation was performed according to a previous publication [21]. Briefly, 4 million cells were seeded onto a pre-coated T75 flask. Twenty four hours later, cells were treated with the differentiation media containing DMEM-F12 with the N2 supplement, 1 μg/mL tetracycline (Merck), 2 ng/mL human recombinant GDNF, and 1 mM dibutyryl cAMP (Santa Cruz, Starr County, TX, USA). After 2 days, cells were trypsinised and replated at 20,000 cells per well in a 96-well plate. These were maintained in the differentiation media and treated on the sixth day after differentiation.

### 2.5. Immunofluorescence

Dopaminergic neurons at day 35 post-differentiation were fixed with 4% *v/v* paraformaldehyde (Sigma) for 10 min at room temperature (RT) before permeabilisation with 0.1% *v*/*v* Tween-20 (Sigma). Cells were then blocked with 5% *v/v* goat serum for 30 min at RT. Staining was performed with primary rabbit anti-tyrosine hydroxylase diluted at 1:1000 (Pel freez, Rogers, AR, USA), primary mouse anti-βIII tubulin at 1:1000 dilution (Abcam, Waltham, MA, USA), and 1:1000 Alexa Fluor 568-conjugated secondary (ThermoFisher Scientific), which was sequentially applied and incubated at RT for 1 h. 4′,6-diamidino-2-phenylindole (DAPI) staining was performed before the images were visualised and imaged using a Nikon Eclipse Ti fluorescent microscope (Nikon, Tokyo, Japan).

### 2.6. STIP1-Derived Peptide Library

The 18-mer peptides with a 13 amino acid shift spanning the entire protein sequence of the human STIP1 were synthesised (Mimotopes, Mulgrave, VIC, Australia). Peptides were used individually or pooled in sets of 5. Lyophilised peptides were dissolved in 200uL of dimethyl sulfoxide (DMSO) to obtain a stock solution of 10 mg/mL.

### 2.7. Production of Full-Length STIP1 Protein

The MultiBac pACEBac1 vector (Geneva Biotech, Geneva, Switzerland) was used to make STIP1 proteins. Recombinant human and mouse STIP1 proteins, each with a C-terminal 6x-His tag were produced in Spodoptera frugiperda (Sf9) cells (UnitProtKB accession numbers P31948 and Q60864). Following standard bacmid preparation and the infection of insect cells, the soluble STIP1 proteins were purified by tandem nickel nitrilotriacetic acid (Thermo Fisher Scientific, Waltham, MA, USA) and size-exclusion column purification (Cytiva, Reugelstraat, Belgium) in 20 mM Tris pH 8.0, 150 mM sodium chloride, and 1 mM dithiothreitol buffer. For storage, STIP1 protein aliquots were sterile filtered and kept in 20% *v*/*v* glycerol at −20 °C.

### 2.8. Primary Hippocampal Neuronal Cell Culture

The animal study was approved by IACUC number 191468. Hippocampal neurons harvested from C57BL/6 mouse embryos at embryonic day 14–15 were dissected and digested using the Papain Dissociation system (Worthington Biochemical Corporation, Lakewood, NJ, USA). Neurons were cultured in neurobasal medium supplemented with B27 (Life Technologies, New York, NY, USA), Glutamax (Life Technologies), penicillin, and streptomycin for 7 days before any treatment. Media were supplemented with cytosine β-D-arabinofuranoside (Sigma-Aldrich) to minimise the glia proliferation. Neurons were then plated at an appropriate density on poly-D-lysine (Sigma-Aldrich) coated plates. Half medium change was performed every 2 to 4 days.

### 2.9. Peptide/Protein-Based ELISA

The STIP1 protein autoantibody and peptide epitope screen were performed using a protein and peptide-based ELISA, as previously described [22]. Briefly, Nunc Maxisorp flat bottom 96-well plates (Invitrogen) were coated with 2 μg/mL of human STIP1 from Sf9 cells diluted in PBS pH 7.2 (Gibco) or 10 μg/mL of peptides. Plates were blocked using 1% *w/v* sodium casein (Merck) diluted in 0.1% *v*/*v* PBST before the addition of plasma from the Parkinson’s disease patients and healthy controls. Plasma samples were diluted 1:1000 in 0.1% *w*/*v* sodium casein. Peroxidase affinipure goat anti-human IgG (Jackson, Lebanon County, PA, USA) at 1:2000 was used as the detecting antibodies. A tetramethylbenizidine (TMB) substrate (Thermo Fisher Scientific) was used for development for 15 min and quenched using 2 M sulphuric acid. Absorbance was read at 450 nm using the Cytation^TM^ 5 cell imaging multi-mode reader (Firmware 3.10.06, Biotek, CA, USA). Absorbance signals were used for the final analysis. Plates were incubated at RT for 1 h for all the steps unless otherwise indicated, and plates were washed thrice with 0.1% *v*/*v* PBST in between steps.

### 2.10. Cell Death Assay

Briefly, hiPSC derived neuronal cells and neuronal cell lines were seeded at 20,000 cells per well and cultured in 96-well plates (Greiner Bio-One, Solingen, Germany) for approximately 24 h prior to the treatment of Sf9 STIP1 and staurosporine (Abcam, Cambridge, UK). After 16–20 h of toxin treatment, cell viability was assessed using the colorimetric CellTiter 96 Aqueous Cell Proliferation Assay (Promega, Madison, WI, USA). The assay converts the 3-(4,5-dimethylthiazol-2-yl)-5-(3-carboxymethoxyphenyl)-2-(4-sulfophenyl)-2H-tetrazolium (MTS) compound into a coloured formazan product in metabolically active cells. A total of 20,000 cells were seeded in triplicate for each condition in 96-well plates. Cells were pre-treated with 1 μM STIP1 protein for 1–2 h prior to the treatment of staurosporine. After 16–20 h in vitro, 20 μL of the MTS assay reagent was added to 100 μL of cell media to each well. Cells were then incubated with the reagent for 2 h before the absorbance measurement at 490 nm using the Infinite M200 plate reader (Firmware V_2.02_11/06, Tecan, Switzerland). Absorbance measurements were used for the final analysis.

### 2.11. T Cell ELISpot

Multiscreen_HTS_ filter Elispot plates (Mabtech, Stockholm, Sweden) were coated with 15 μg/mL of human IFNγ antibody (1-DIK) overnight at 4 °C. A total of 100,000 PBMCs were placed in each well and stimulated with 5 μg/mL of pooled and/or individual STIP1 peptides from the aforementioned peptide library for 18 h with 5 IU/mL of IL-2. The CD3/CD28/CD2 T cell activator (STEMCELL Technologies, Vancouver, BC, Canada) was used as the positive control. The plates were then developed using the 1:1000 human biotinylated IFNγ detection antibody (7-B6–1), followed by streptavidin ALP and BCIP/NBT phosphatase substrate (Sigma Aldrich). The number of spot forming units (SFU) were quantified using the Mabtech Iris^TM^ FluoroSpot/ELISpot reader system equipped with Spot reader software version 1.1.9 and included in the analysis. Out of the 15 individuals with high STIP1 autoantibodies, seven individuals returned for the follow-up and were recruited for the characterisation of STIP-specific T cells.

### 2.12. Cancer Outlier Profile Analysis (COPA)

Assay readings from the STIP1 protein and peptide-based ELISAs were background subtracted and normalised for batch-to-batch variation by median centering. Outliers were then detected via a robust non-parametric method according to Tomalin et al. [23] where the normalised readings were median centred and scaled by the MAD (median absolute deviation). Outliers were defined as samples with a signal greater than the 75th percentile of the data plus the 1.5 interquartile range (IQR).

### 2.13. Statistical Analysis

Data are presented as the mean ± standard error of the mean (SEM) of three independent experiments unless otherwise stated. The paired *t*-test was performed for a comparison between different treatment groups using GraphPad Prism 8.2 software (GraphPad Software, San Diego, CA, USA). A value of *p* < 0.05 was considered as statistically significant.

## 3. Results

### 3.1. STIP1 Ameliorates Staurosporine-Induced Neurotoxicity on Dopaminergic Neurons Derived from hiPSC

**Figure 1:**
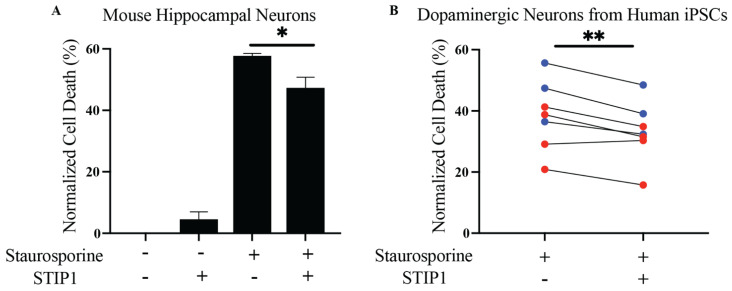
Human STIP1 ameliorates staurosporine-induced neurotoxicity in human dopaminergic neurons derived from induced pluripotent stem cells. Neurons were pre-treated with STIP1 over 1 h prior to staurosporine treatment over 16–20 h. Cell viability was quantified using the MTS assay and bar graphs are represented as the mean ± standard error of mean (SEM) percentage of cell death normalised against untreated neurons for each condition. (**A**) Primary mouse hippocampal neurons were treated with 1 μM mouse STIP1 followed by 31.25 nM staurosporine treatment. (**B**) Human induced pluripotent stem cells (hiPSCs) derived dopaminergic neurons from healthy (blue) and Parkinson’s disease (red) individuals were treated with 1 μM human STIP1 followed by 250 nM staurosporine. All experiments were independently repeated two to four times. Paired *t*-test analysis was performed for all samples except for the primary mouse hippocampal neurons, which was analysed using an unpaired *t*-test. * *p* < 0.05, ** *p* < 0.01.

STIP1 plays important roles in embryonic development, enhancing neuronal resilience, neuronal differentiation, and protein synthesis [10]. More importantly, STIP1 was found to mitigate staurosporine-induced neurotoxicity and β-amyloid toxicity in mouse primary hippocampal neurons [11,13]. However, the functional significance of STIP1 on dopaminergic neurons has yet to be examined. We produced recombinant human and mouse STIP1 by using an insect cell-based expression system. Next, to assess STIP1’s putative protective effect and test the sensitivity of our experimental readout, we first reproduced Beraldo’s observations on the alleviation of staurosporine-induced neurotoxicity on mouse primary hippocampal neurons [11] (Figure 1A). Staurosporine acts as a non-selective protein kinase inhibitor to induce apoptosis [24]. In line with the expectations, the exogenous addition of STIP1 in the supernatant resulted in a small but significant decrease in cell death. Next, to evaluate the protective effect on Parkinson’s disease-related neurons, we generated dopaminergic neurons from the HC and Parkinson’s disease individuals’ iPSCs. The dopaminergic neuronal phenotype of these hiPSC derived neurons was validated through the expression of tyrosine hydroxylase and βIII tubulin (Figure S1). Similarly, a significant reduction in staurosporine-induced cell death was observed in the presence of STIP1 (*p* = 0.0047, two-tailed, paired *t*-test) (Figure 1B). This trend was reproduced in both the Parkinson’s disease patient and HC derived neurons. Thus, STIP1 seems to enhance the neuronal resilience of dopaminergic neurons.

### 3.2. STIP1-Specific Autoantibodies in Parkinson’s Disease Patients and Healthy Controls

**Figure 2:**
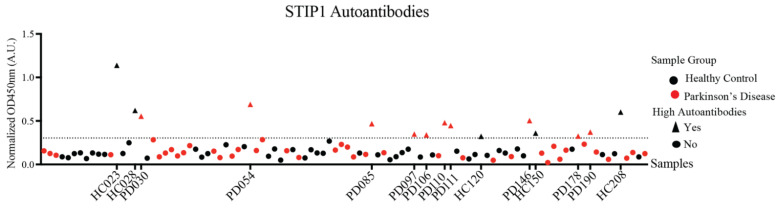
STIP1 autoantibody titres in the Parkinson’s disease patients and healthy controls. Plasma samples from the Parkinson’s disease (PD) patients and healthy controls (HC) (*n* = 50 per group, 1:1000 dilution) were subjected to an STIP1 protein-based ELISA assay. Each data point represents the averaged normalised signal after the median centering of signals from various batches for each individual. Using the COPA outlier analysis, the dotted threshold line defines samples above the 75th percentile of the data plus the 1.5 interquartile range to have high autoantibodies. Samples with high autoantibodies are indicated as triangles while samples with low autoantibodies are represented by circles. Parkinson’s disease patient samples are represented in red while healthy controls are represented in black. Sample codes of Parkinson’s disease patients and healthy controls identified to have high STIP1 autoantibodies are listed on the *x*-axis.

STIP1 autoantibodies were previously reported in the mothers of ASD children and present at high levels in neuro-Behçet’s disease [17,18]. Given STIP1’s role in brain development and its neuroprotective role in neurological disorders, we posit that the presence of autoantibodies against the STIP1 protein may contribute to Parkinson’s disease pathogenesis, predisposing individuals to Parkinson’s disease development. To determine the presence of STIP1 autoantibodies, a STIP1 protein-based ELISA was performed using plasma samples from 50 patients and 50 age-matched HCs (the demographic information is listed in Table 1). STIP1-specific autoantibodies were detected in both the Parkinson’s disease patients and HCs (*p* = 0.25, two-tailed, Mann–Whitney). However, a cancer outlier profile analysis (COPA) revealed more patients (*n* = 10, 20%) with higher levels of autoantibodies compared to the HCs (*n* = 5, 10%) (*p* = 0.26, Fischer’s exact) (Figure 2).

**Figure 3:**
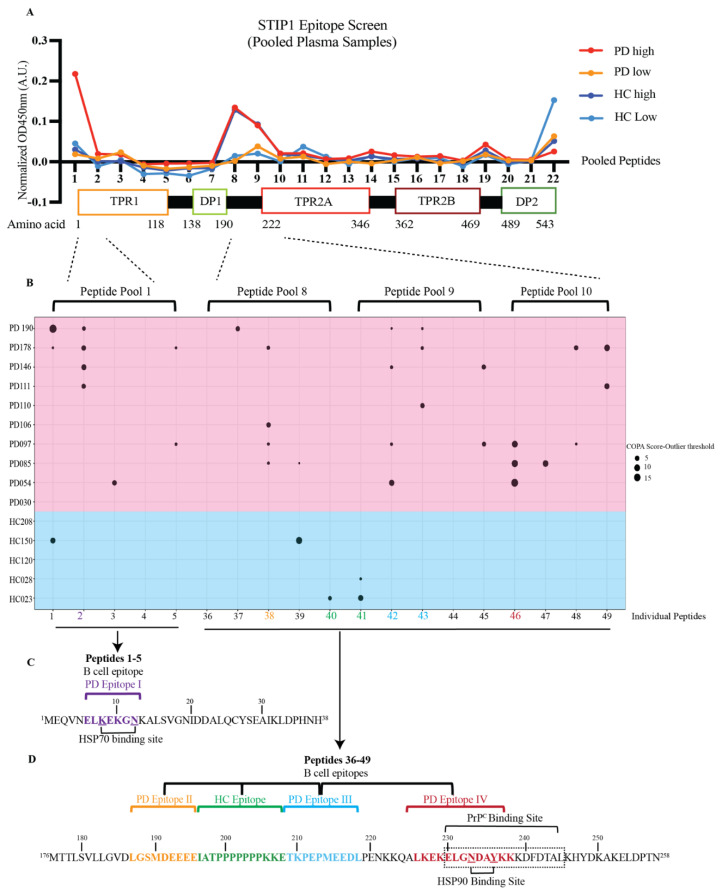
STIP1 autoantibody binding patterns in Parkinson’s disease patients and healthy controls. (**A**) A 22-pooled peptide-based ELISA with each pool containing 5 peptides was performed. Pooled plasma samples of the healthy controls (HC) and Parkinson’s disease (PD) patients according to the levels of the STIP1 autoantibodies were made and screened against the pooled peptides. A linear representation of STIP1 domains that coincide with the pooled peptides and their respective amino acid number is shown. (**B**) A COPA table highlighting the distribution of the STIP1 autoantibody reactivity against individual peptides within pool 1 (peptides 1–5), pool 8 (peptides 36–40), 9 (peptides 41–45), and 10 (peptides 46–49) using the healthy control (Blue background) and Parkinson’s disease (red background) samples with high autoantibodies. A positive signal was determined using the difference between the COPA score and the COPA threshold. The size of the circles revealed the distance between the COPA threshold and the COPA score. A bigger circle coincides with a higher reactivity against the peptide. Amino acid sequences of (**C**) peptides 1–5 and (**D**) peptides 36–49 are shown. The core B cell epitope of the four PD epitopes I, II, III, IV are coded in purple, orange, blue and red, respectively. The HC epitope is coded in green. The underlined amino acids represent regions that associate to the HSP70 and HSP90 binding sites while the amino acids that are boxed up highlight the PrP^C^ binding site.

Fine mapping of the recognised epitopes further revealed substantial differences in the recognition patterns of patients and HCs (Figure 3). STIP1 has three tetratricopeptide repeat (TPR) domains (TPR1, TPR2A, and TPR2B) and two aspartate and proline-rich domains (DP1 and DP2) (Figure 3A). These domains are involved in the co-chaperone function and PrP^C^ ligation. To elucidate the STIP1 autoantibody’s binding profile, we used a peptide library containing 18 amino acid (aa) long peptides with a 13aa overlap from the full-length STIP1 protein (UniProtKB accession number P31948). For the initial screen, peptide pools containing five peptides were prepared to analyse the pooled plasma samples from the HCs and patients with either high- or low-levels of the STIP1 autoantibodies. The analysis revealed that the *N*-terminus at TPR1 (peptide pool 1) and the region between DP1 and TPR2A (peptide pools 8–10) were preferably recognised by the plasma derived from Parkinson’s disease patients with high autoantibody levels against the STP1 protein (Figure 3A).

To further narrow down the binding sites of the autoantibodies, we screened the plasma samples of the 10 Parkinson’s disease patients and five HCs previously defined by the COPA to have high levels of STIP1 autoantibodies (see Figure 2), with the individual peptides from pool 1 and pools 8–10 (Figure 3B). Another COPA was carried out to filter out the true binding signals from noise. The COPA delineated about four Parkinson’s disease-specific epitopes. The first B cell epitope (PD epitope I) targets peptide 2 (aa 6–23), containing a reported binding site of HSP70. The second (PD epitope II) and the third (PD epitope III) epitopes overlap with peptide 38 (aa 186–203) and peptides 42/43 (aa 206 to 228), respectively. This region flanks a flexible hinge region between DP1 and TPR2A. The region covering peptide 38 was previously reported in mothers with ASD children [25]. The fourth epitope (PD epitope IV) covers peptide 46 (aa 226–243) (Figure 3B), which coincides with the HSP90 and PrP^C^ binding sites [12,26,27]. The core B cell epitope of PD epitopes I, II, III, IV were further defined as ELKEKGN (aa 6–13), LGSMDEEEE (aa 187–195), TKPEPMEEDL (aa 209–218), and LKEKELGNDAYKK (aa 226–238), respectively (Figure 3C,D). In contrast to the patients, only a few HCs displayed peptide-specific binding. This was evident from the detection of two out of four HC epitopes recognising peptide 40/41 (HC epitope spanning IATPPPPPPPKKE, aa196–208), a region not recognised by the Parkinson’s disease samples (Figure 3D).

### 3.3. STIP1-Specific T Cell Epitopes Cluster around the PrP^C^ Binding Site

**Figure 4:**
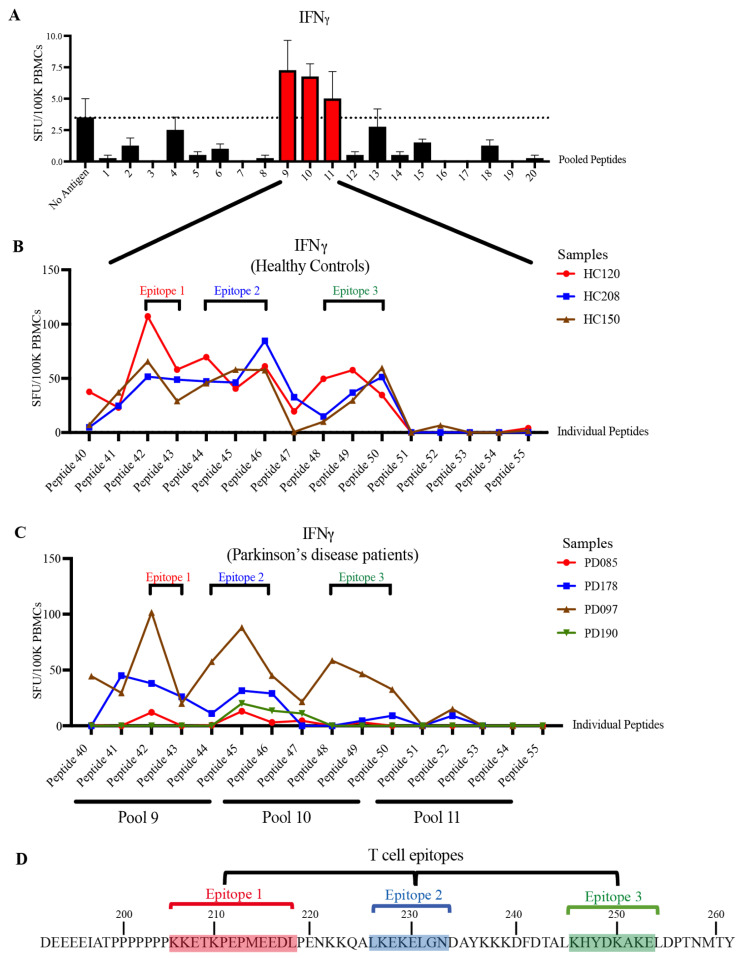
Characterisation of STIP1-specific T cell epitopes. PBMCs obtained from (**A**) healthy controls (HC) were stimulated with 5 μg/mL of pooled peptides. Each pool contains 5–6 individual peptides from the STIP1 peptide library. The dotted line represents the average spot forming units (SFU) from the background, which are cells without antigen treatment. The SFU of interferonγ (IFNγ)-secreting cells per 100,000 (100 K) PBMCs is shown. The T cell epitope characterisation was performed by activating (**B**) HC (*n* = 3) and (**C**) Parkinson’s disease (PD) (*n* = 4) PBMCs with individual peptides (peptides 41–55) from pooled peptides 9, 10, and 11. Three core epitopes were defined. Each dot represents the SFU of antigen stimulation after the deduction of SFU from the condition without antigen stimulation. (**D**) Epitope 1 (red) comprises peptides 42–43, epitope 2 (blue) consists of peptides 44–46, and epitope 3 (green) includes peptides 48–50. An illustration of the core epitopes 1, 2, and 3 are highlighted in red, blue, and green, respectively.

For humoral immune responses to be activated, signals from an antigen-specific helper T cell are required [28]. Screening of the patient and HCs’ PBMCs with the STIP1 protein revealed the presence of autoreactive STIP1-specific T cells in a subset of individuals (Figure S2). When pooled fractions of the STIP1 peptide library was used, T cell reactivity was found to be predominantly directed against peptide pools 9, 10, and 11 (Figure 4A). Further examination of the T cell epitope using individual peptides revealed approximately three immunodominant epitopes recognised in both the HCs and Parkinson’s disease patients (Figure 4B,C). In contrast to the autoantibody epitopes, there were no substantial differences in the specificity of the T cell response between the Parkinson’s disease patients and HCs. T cell epitopes 1, 2, and 3 span aa 206–218, 226–233, and 246–253, respectively. Based on the recognition of overlapping peptides, their core epitopes were defined as KKETKPEPMEEDL (epitope 1), LKEKELGN (epitope 2), and KHYDKAKE (epitope 3) (Figure 4D). These are all located proximal to the PrP^C^ binding site, a region predominantly recognised by autoantibodies (Figure 3).

### 3.4. STIP1 Autoantibody Binding Sites Overlap with the HSP70 and PrP^C^ Association Regions

**Figure 5:**
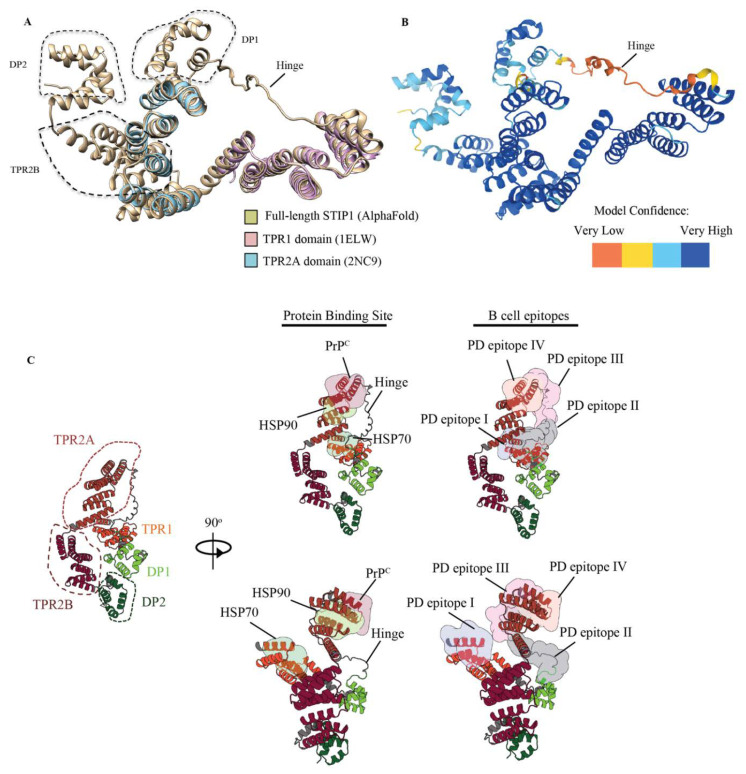
Architecture of the human STIP1 showing canonical protein binding sites and B cell epitopes. (**A**) Structural alignment of human stip1 (AlphaFold) and known high-resolution structures of the human TPR1 and TPR2A domains. The structure shows an overall root mean square deviation of 117 pairs of 0.58Å. Alignment using the X-ray structure PDB ID: 1ELW and nuclear magnetic resonance structure PDB ID: 2NC9 was performed. The molecular analysis was conducted using the UCSF Chimera software [30]. (**B**) The hinge region of the STIP1 protein spanning aa 187 to 217 had a low confidence score, indicating that it may be unstructured in isolation. (**C**) Cartoon representation of the full-length human STIP1 protein predicted using AlphaFold. The protein binding sites of HSP70, HSP90, prion (PrP^C^), and the Parkinson’s disease autoantibody epitopes on STIP1 are shown with coloured surfaces according to the labels. Illustrations were drawn with Protein Imager [31].

While nuclear magnetic resonance and X-ray crystallography data of the TPR1 and TPR2A domains exist, the overall structure of STIP1 has not been resolved. AlphaFold is the latest artificial intelligence system that predicts a protein’s 3D structure with high accuracy from its amino acid sequence [29]. STIP1 derived from yeast was previously reported to be an elongated structure [26]. Using AlphaFold, we generated a structure of the full-length STIP1 that was in excellent agreement with the elucidated structures of the TPR1 and TP2A domains (Figure 5A). With the exception of the highly flexible linker region, the model confidence was in fact mostly very high (Figure 5B). The PrP^C^ and HSP70 association sites on STIP1 were in close proximity to this hinge. These regions were also spatially positioned near the HSP90 binding site but away from the C-terminal DP2 domain. In contrast, DP1 and TPR2B were clustered closer to DP2 (Figure 5C). In this work, we highlighted four major B cell epitopes recognised by the autoantibodies of Parkinson’s disease patients (PD epitopes I, II, III, IV). As illustrated in the 3D model, they target the regions of the protein that serve as binding sites for HSP70, HSP90, and PrP^C^ as well as the flexible hinge region. Unlike PD epitopes I, III, and IV, which all localise in the helical-rich regions, the PD epitope II lies within the unstructured hinge (Figure 5). The region spanning PD epitope II was previously noted to be highly dynamic and is consistent with the low confidence score in the AlphaFold model (Figure 5B) [29]. It is noteworthy that the hinge contains a rigid poly-proline rich region (aa 199–205) that loops across the PrP^C^ binding site. The PD epitope I shares two aa residues, Lys8 and Asn12, on the STIP1’s TPR1 domain that is associated with the HSP70 peptide (Figure 3C) [27]. The PD epitope III lies adjacent to the PrP^C^ binding site occupying TPR2A and the hinge region. The PD epitope IV overlaps with the PrP^C^ binding domain, and two aa, Asn233 and Tyr236, that are associated with the HSP90 peptide (Figure 3D) [27]. Altogether, the presence of STIP1 autoantibodies may disrupt the STIP1-to-PrP^C^ engagement and the STIP1’s role as a co-chaperone when it associates with HSP70, possibly increasing an individual’s risk of the development of Parkinson’s disease.

## 4. Discussion

The present study provides the first experimental evidence of STIP1’s neuroprotective effect on dopaminergic neurons and examines how immune dysregulation resulting in the formation of STIP1-specific autoantibodies may predispose individuals to Parkinson’s disease development.

The role of STIP1 in Parkinson’s disease is not well understood. Hence, an assessment of the STIP1 functionality in Parkinson’s disease was performed using hiPSC derived dopaminergic neurons. We reproduced the neuroprotective effect of mouse STIP1 on staurosporine treated mouse hippocampal neurons and observed STIP1’s ability to rescue dopaminergic neurons derived from hiPSCs. This effect was not observed with the use of SH-SY5Y cells and LUHMES human dopaminergic cell lines (Figure S3), highlighting the importance of using experimental models that are physiologically similar to the neurons in the brain.

Screening of plasma samples with recombinant STIP1 protein revealed high levels of STIP1-specific autoantibodies in about 20% of Parkinson’s disease patients and 10% of HCs. The detailed characterisation of the B cell epitopes further revealed striking differences between the two groups. For Parkinson’s disease patients, four immunodominant regions were identified. One of these regions (B epitope IV) overlapped the well-characterised PrP^C^ binding site on STIP1, spanning aa 230–245 [12]. Antibodies against this region were previously reported to reduce STIP1 mediated neuritogenesis in primary mouse hippocampal neurons, underscoring the importance of STIP1-PrP^C^ interaction [10]. PrP^C^ is also a critical player in the induction of soluble protein aggregates such as β-amyloid, α-synuclein, and the tau protein [32]. Significantly, α-synuclein, which is involved in Parkinson’s disease, was discovered to bind to PrP^C^, compromising hippocampal neuronal function and structure [32,33,34]. PrP^C^ also mediates α-synuclein cell-to-cell spreading, evident from an increased uptake of α-synuclein amyloids in in vitro PrP^C^ overexpressing cells, and in vivo wild-type PrP^C^ expressing mouse models [35]. The region spanning aa 93–109 on the PrP^C^ was found to be essential for the α-synuclein oligomer mediated long-term potentiation inhibition [34]. Given that STIP1 binds to the aa 113–128 on the PrP^C^, it is worth investigating whether STIP1’s association to PrP^C^ may competitively interfere with α-synuclein binding to PrP^C^ [12]. The inferred interference of STIP1’s association with PrP^C^ by autoantibodies may thus have implications on STIP1’s neuroprotective capacity through both signalling pathways as well as its ability to competitively interfere with the effects of disease associated proteins such as α-synuclein and β-amyloid [10,14].

STIP1 is a co-chaperone with cardinal roles against proteotoxicity. Wolfe et al. [36] showed that the absence of STIP1 exacerbated Huntingtin with 103Q glutamine stretch (Htt103Q) toxicity while STIP1 elevation suppressed the Htt toxicity in yeast. Conversely, the knockdown of STIP1 reduced mutant Huntingtin aggregation and toxicity in a drosophila model [37]. The neuroprotective role of STIP1 remains controversial as a new study by Lackie et al. [38] showed an increased amyloid burden with amplified neurotoxicity in the presence of elevated STIP1 using their Alzheimer’s mouse model. This observation was in contrast to STIP1’s neuroprotective effect on in vitro mouse hippocampal neurons and in Caenorhabditis elegans [38]. Notably, the neuroprotection present in the STIP1 overexpression mouse model against exogenous amyloid-β neurotoxicity in vitro was due to extracellular STIP1, suggested from the neutralisation of neuroprotection with anti-STIP1 antibodies [38]. These studies imply that STIP1’s role in proteostasis may be different in vitro and in vivo according to the neuronal subtype studied and animal model used. It is also possible that STIP1 may not be effective alone as STIP1 failed to prevent α-synuclein elongation by itself but was effective in the presence of HSP90 [39]. Finally, STIP1’s knockout in human cell lines unexpectedly displayed improved protein folding despite the proteasomal defect by enhancing HSP70-HSP90’s folding capacity. This suggests that STIP1 in eukaryotes shifts the proteostatic balance to enable a greater reliance on proteasomal degradation instead of refolding [40].

**Figure 6:**
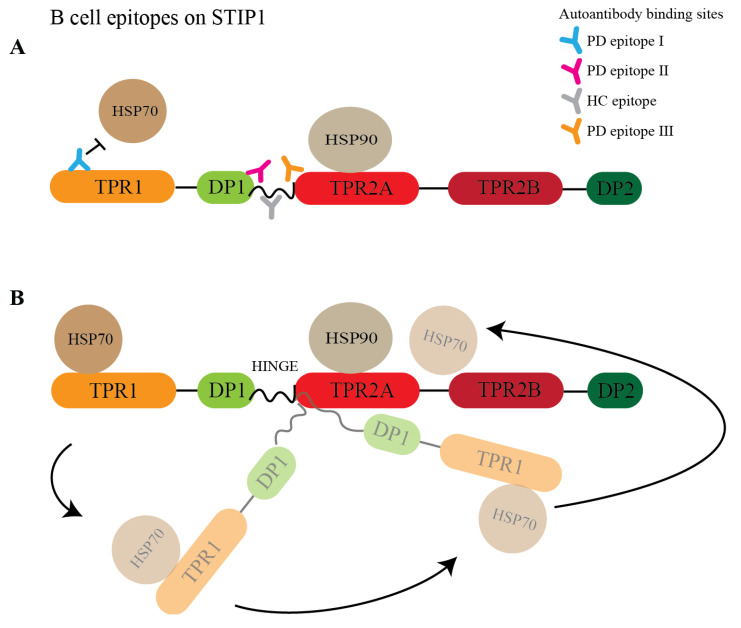
Model of the STIP1 autoantibodies disrupting the chaperone machinery. (**A**) STIP1 autoantibodies targeting various domains are shown. Parkinson’s disease antibodies recognise peptide 2 (PD epitope I), 38 (PD epitope II), and 42/43 (PD epitope III) while healthy control-specific antibodies target peptides 40/41 (HC epitope). The presence of autoantibodies will disrupt the association of HSP70s with TPR1 and (**B**) impede the function of the flexible hinge that is crucial for the HSP70s’ interdomain movement from TPR1 to TPR2B. This movement allows HSP70 to be in close proximity to HSP90 for client transfer and maturation.

The primary role of STIP1 as a co-chaperone involves client protein transfer from HSP70 to HSP90 (Figure 6B). Notably, three of the four epitopes recognised by autoantibodies in Parkinson’s disease patients targeted regions related to this function. PD epitope I recognised the *N*-terminal region of STIP1, which coincides with the C-terminal HSP70 binding region. While this may hinder HSP70’s binding to STIP1’s TPR1 domain, autoantibodies against the flexible hinge (PD epitope II and III) might interfere with HSP70’s interdomain movement [27]. This hinge region plays a key role as it facilitates the interdomain translocation of HSP70 from TPR1 to TPR2B, enabling HSP70 to be proximal to HSP90. Röhl et al. [26] demonstrated that the linker regulates client activation (e.g., activation of the glucocorticoid receptor) and modulates HSP70 binding to different domains in the presence of HSP90. This led to the proposal that the linker is responsible for HSP70’s movement from the TPR1 to the TPR2B domain, enabling client transfer and activation when HSP90 binds to the TPR2A domain [26]. The B cell epitopes defined by the PD epitopes II and III target the interface between the linker and domains, suggesting that the autoantibody binding may affect client refolding, thereby increasing aggregated protein formation, which is a hallmark of Parkinson’s disease. Of note, autoantibodies detected in the two HCs (HC epitope) predominantly targeted the proline-rich middle region of the hinge (IATPPPPPPPKKE) while the Parkinson’s disease antibodies targeted the more flexible flanking regions (Figure 6A,B).

Currently, our understanding of co-chaperones and HSPs is confined to its intracellular role. However, the role of extracellular chaperones in neurodegenerative conditions is relevant in the context of autoantibody studies. Extracellular HSP70s have been studied in Alzheimer’s disease and was found to be as effective as cytosolic HSP70 in preventing amyloid β42 (Aβ42)-induced neuronal death in drosophila models [41]. Interestingly, the mechanism of extracellular HSP70s’ neuroprotection differs slightly from intracellular HSP70 as it works primarily by sequestering Aβ42 through its holdase activity, thereby masking Aβ42 neurotoxicity [42]. Clusterin is another extracellular chaperone reported to bind and regulate the amyloid-β-neurotoxic effect [43]. Recently, Foster et al. [44] showed that clusterin enhances Tau aggregate seeding, exacerbating Tau pathology in Alzheimer’s disease. Thus, extracellular chaperones have varied roles in modulating neuronal susceptibilities to aggregated proteins. Although the effects of extracellular HSPs and STIP1 in the context of Parkinson’s disease remain unclear, its extracellular interaction has been documented to be essential for extracellular matrix remodelling and subventricular zone neuroblast migration [45,46]. With evidence of STIP1’s association with HSPs extracellularly, Parkinson’s disease-specific STIP1 autoantibodies may potentially impede the concerted effects of extracellular STIP1 and HSPs.

Previous reports on STIP1 autoantibodies in the mothers of ASD children further support the detrimental effects these autoantibodies may have on neurodevelopment [25]. Strikingly, we detected autoantibodies against aa 185 to 199 overlapping with PD epitope II, one of our B cell epitopes targeting the hinge region. The autoimmune response against the extracellular function of STIP1 in the PrP^C^-mediated signalling pathways and the chaperone machinery may therefore predispose individuals to neurological diseases beyond Parkinson’s disease. However, our study identified these autoantibodies only in a subset of patients. Given the complex nature of the pathogenesis of Parkinson’s disease, the contribution of autoimmune mechanisms may occur only in individuals with an autoimmune predisposition. Aberrant immune responses against endogenous proteins have been previously reported in Parkinson’s disease patients. This is evident from the identification of α-synuclein-specific T cells in patients, mitochondrial-specific CD8+ cells in PTEN-induced putative kinase 1 knockout (PINK−/−) mice, and CD8+ T cells infiltrating the SNc prior to neuronal death and α-synuclein aggregation [47,48,49]. Furthermore, age-related perturbations of the immune system from immunosenescence, inflammaging, and the decline in adaptive immune cells may predispose individuals to an-age acquired autoimmunity, resulting in the production of autoreactive immune cells [50]. Alternatively, pathogenic processes preceding or occurring during Parkinson’s disease may cause endogenous proteins to be recognised as foreign antigens. We also described the potential of STIP1 as an endogenous neuroprotective agent in Parkinson’s disease. Further in vivo studies to decipher the role of STIP1 autoantibodies by investigating its interaction with the STIP1–PrP^C^ complex, the chaperone machinery, and its downstream immunomodulatory effects may unravel novel therapeutic targets for Parkinson’s disease.

## Figures and Tables

**Table 1:** Demographics of the participants.

Clinical Parameters	Healthy Controls	Parkinson’s Disease Patients
Age (mean ± standard deviation)	63.1 (±8)	62.94 (±8.48)
Gender (Sample size)	Males, *n* = 27 Females, *n* = 23	Males, *n* = 27 Females, *n* = 23
Hoehn and Yahr Staging (%)	Not applicable	≤2 (45%) >2 (55%)
Ethnicity (Sample size)	Chinese, *n* = 50	Chinese, *n* = 48 Eurasian, *n* = 1 Indian, *n* = 1

## Data Availability

Not applicable.

## Supplementary Materials

The following supporting information can be downloaded at: https://www.mdpi.com/article/10.3390/cells11101649/s1, Figure S1: Characterisation of human iPSCs derived dopaminergic neurons. Figure S2: STIP1-specific T cell responses in healthy controls and Parkinson’s disease patients; Figure S3: Human STIP1 did not rescue staurosporine-induced neurotoxicity in human SH-SY5Y cells and LUHMES dopaminergic cell lines.
