# Supplementary material for: Parkinson’s Disease-Specific Autoantibodies against the Neuroprotective Co-Chaperone STIP1
Source: Cells. 2022 May 16;11(10):1649. doi: 10.3390/cells11101649 (PMC9139896; doi:10.3390/cells11101649)
Supplement: Supplementary file 1 [file cells-11-01649-s001.zip › cells-1697262-supplementary.pdf]

Supplementary Material

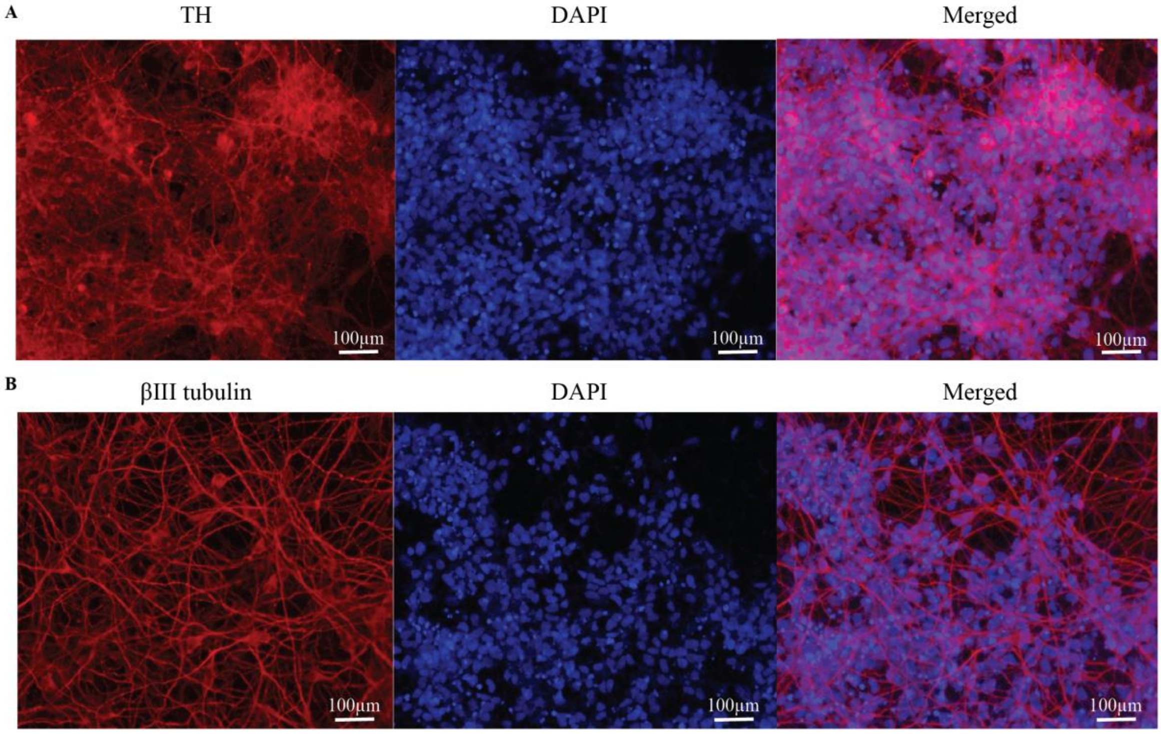

Supplementary Figure S1: Characterization of human iPSCs derived dopaminergic neurons. Dopaminergic neurons at day 35 post-differentiation were stained with (A) anti-tyrosine hydroxylase (TH) (red) the enzyme in the catecholamine synthetic pathway, (B) anti-βIII tubulin (red) a neuronal marker, and 4',6-diamidino-2-phenylindole (DAPI) (Blue) for the nucleus.

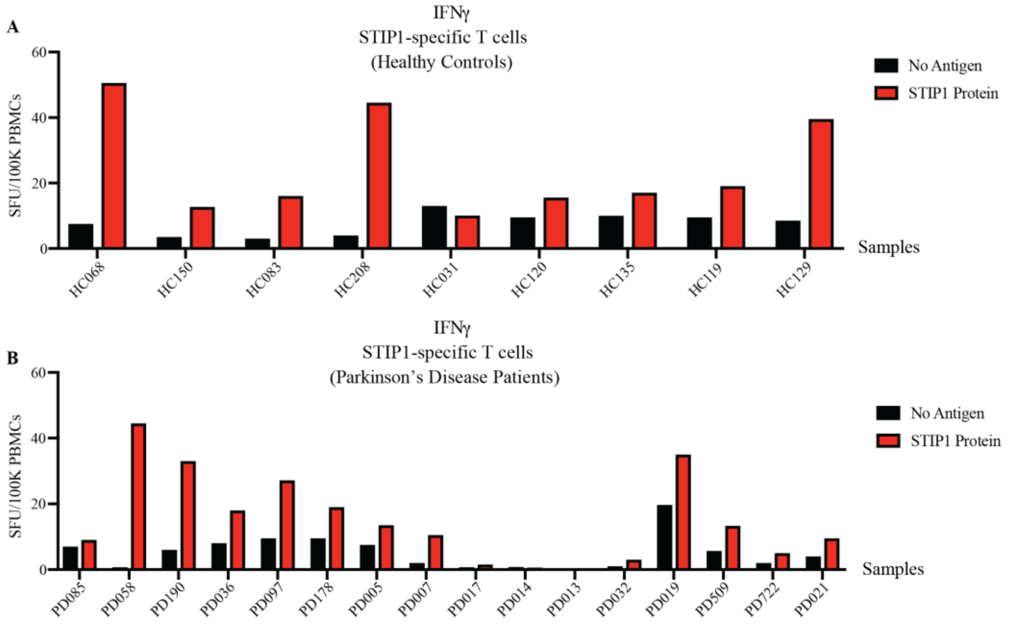

Supplementary Figure S2: STIP1-specific T cell responses in healthy controls and Parkinson's disease patients. PBMCs obtained from (**A**) Healthy controls (HC) (*n*=9) and (**B**) Parkinson's disease (PD) patients (*n*=16) were stimulated with 5ug/ml of STIP1 protein. STIP1-specific T cell responses were detected from the number of spot-forming units (SFU) of interferonγ (IFNγ)-secreting cells per 100 000 (100K) PBMCs. The condition without antigen stimulation is the background signal.

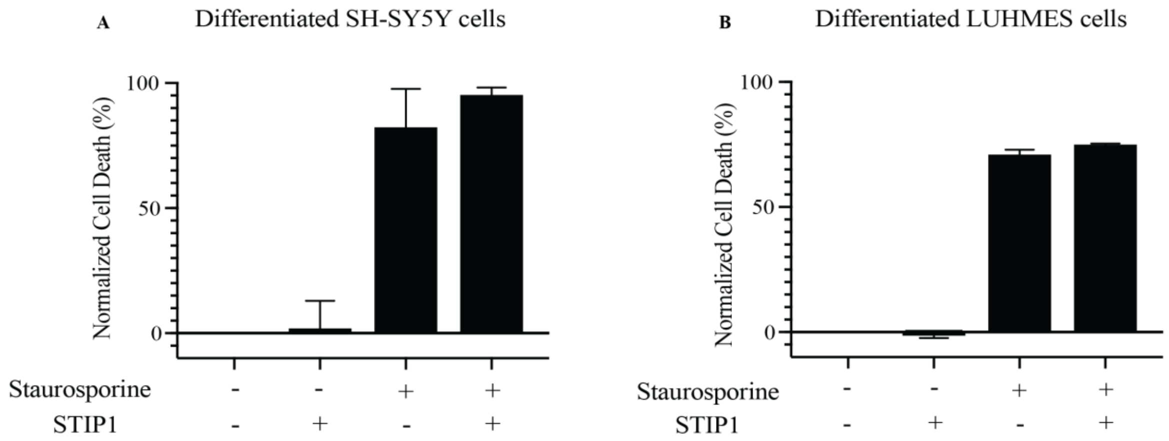

Supplementary Figure S3: Human STIP1 did not rescue staurosporine-induced neurotoxicity in human SH-SY5Y cells and LUHMES dopaminergic cell lines. Differentiated (**A**) SH-SY5Y cells and (**B**) LUHMES neurons were pretreated with 1uM STIP1 for 1 hour prior to 250nM staurosporine treatment over 16-20 hours. Cell viability was quantified using the MTS assay and bar graphs are represented as the mean ± standard error of mean (SEM) percentage of cell death normalized against untreated neurons for each condition.
